# Vaccines for preventing herpes zoster in older adults

**DOI:** 10.1590/1516-3180.20141324T1

**Published:** 2014-05-20

**Authors:** Ana M. Z. Gagliardi, Brenda Nazaré Gomes Silva, Maria Regina Torloni, Bernardo Garcia de Oliveira Soares

## Abstract

**BACKGROUND:**

Herpes zoster or, as it is commonly called, ‘shingles’ is a neurocutaneous disease characterised by the reactivation of varicella zoster virus (VZV), the virus that causes chickenpox, which is latent in the dorsal spinal ganglia when immunity to VZV declines. It is an extremely painful condition which can often last for many weeks or months, impairing the patient’s quality of life. The natural aging process is associated with a reduction of cellular immunity which predisposes to herpes zoster. Vaccination with an attenuated form of VZV activates specific T cell production, therefore avoiding viral reactivation. A herpes zoster vaccine with an active virus has been approved for clinical use among older adults by the Food and Drug Administration and has been tested in large populations.

**OBJECTIVE:**

To evaluate the effectiveness and safety of vaccination for preventing herpes zoster in older adults.

**METHODS:**

*Search methods:* We searched the following sources for relevant studies: CENTRAL 2012, Issue 7, MEDLINE (1948 to July week 1, 2012), EMBASE (2010 to July 2012), LILACS (1982 to July 2012) and CINAHL (1981 to July 2012). We also reviewed reference lists of identified trials and reviews for additional studies.

*Selection criteria:* Randomised controlled trials (RCTs) or quasi-RCTs comparing zoster vaccine with placebo or no vaccine, to prevent herpes zoster in older adults (mean age > 60 years).

*Data collection and analysis:* Two review authors independently collected and analysed data using a data extraction form. They also carried out an assessment of risk of bias.

**MAIN RESULTS:**

We identified eight RCTs with a total of 52,269 participants. Three studies were classified at low risk of bias. The main outcomes on effectiveness and safety were extracted from one clinical trial with a low risk of bias. Four studies compared zoster vaccine versus placebo; one study compared high-potency zoster vaccine versus low-potency zoster vaccine; one study compared refrigerated zoster vaccine versus frozen zoster vaccine; one study compared live zoster vaccine versus inactivated zoster vaccine and one study compared zoster vaccine versus pneumococcal polysaccharide vaccine (pneumo 23).

Confirmed cases of herpes zoster were less frequent in patients who received the vaccine than in those who received a placebo: risk ratio (RR) 0.49 (95% confidence interval (CI) 0.43 to 0.56), with a risk difference (RD) of 2%, and number needed to treat to benefit (NNTB) of 50. Analyses according to age groups indicated a greater benefit in participants aged 60 to 69 years, RR 0.36 (95% CI 0.30 to 0.45) and in participants aged 70 years and over, RR 0.63 (95% CI 0.53 to 0.75). Vaccine-related systemic adverse effects were more frequent in the vaccinated group (RR 1.29, 95% CI 1.05 to 1.57, number needed to treat to harm (NNTH) = 100). The pooled data risk ratio for adverse effects for participants with one or more inoculation site adverse effect was RR 4.51 (95% CI 2.35 to 8.68), and the NNTH was 2.8 (95% CI 2.3 to 3.4). Side effects were more frequent in younger (60 to 69 years) than in older (70 years and over) participants.

**AUTHORS’ CONCLUSIONS:**

Herpes zoster vaccine is effective in preventing herpes zoster disease. Although vaccine benefits are larger in the younger age group (60 to 69 years), this is also the age group with more adverse events. In general, zoster vaccine is well tolerated; it produces few systemic adverse events and injection site adverse effects of mild to moderate intensity.

**This is the abstract of a Cochrane Review published in the Cochrane Database of Systematic Reviews (CDSR) 2012, issue 10. Art. No.: CD008858**. DOI: 10.1002/14651858.CD008858.pub2 (http://onlinelibrary.wiley.com/doi/10.1002/14651858.CD008858.pub2/full). For full citation and authors’ details, see reference 1.

**The full text is available from:** http://onlinelibrary.wiley.com/doi/10.1002/14651858.CD008858.pub2/pdf

**The abstract is also available in the Portuguese, French, Spanish and Russian languages from:** http://onlinelibrary.wiley.com/doi/10.1002/14651858.CD008858.pub2/abstract.
